# Expert Consensus on the Diagnosis and Management of Carotid Atherosclerotic Plaque: Pathophysiology, Clinical Management, and Preventive Approaches

**DOI:** 10.7150/ijms.107479

**Published:** 2025-05-30

**Authors:** Xing Chang, Hang Zhu, Zhijiang Guo, Hongshuo Shi, Yingjie Tian, Qianying Hao, Hui Zhang, Rongjun Zou, Xiaoping Fan, Qihui Zhang, Sang-Bing Ong, Qingyong He, Hao Zhou

**Affiliations:** 1 Guang'anmen Hospital, China Academy of Chinese Medical Sciences, Beijing, 100053; 2 The Sixth Medical Center of PLA General Hospital, Beijing, 100048; 3 Shuguang Hospital, Shanghai University of Traditional Chinese Medicine, Shanghai, 201203; 4 Beijing University of Chinese Medicine, Beijing, 100029; 5 Hubei University of Chinese Medicine, Hubei, Wuhan, 430065; 6 State Key Laboratory of Traditional Chinese Medicine Syndrome/Department of Cardiovascular Surgery, Guangdong Provincial Hospital of Chinese Medicine, the Second Affiliated Hospital of Guangzhou University of Chinese Medicine, the Second Clinical College of Guangzhou University of Chinese Medicine, Guangzhou 510120, Guangdong, China.; 7 Dongfang Hospital, Beijing University of Chinese Medicine, 100078

**Keywords:** Consensus, Carotid atherosclerosis with plaque, Cholesterol, Medication therapy management

## Abstract

To standardize and harmonize pharmacist-led cholesterol-lowering medication therapy management (MTM) for patients with carotid atherosclerosis and plaque, the Expert Consensus on Lipid-Lowering Pharmacotherapy Management in Patients with Carotid Atherosclerosis and Plaque was developed under the leadership of the PLA General Hospital. This consensus establishes a systematic framework spanning the full-cycle management process: data collection, analytical evaluation, intervention implementation, and long-term follow-up, supported by standardized protocols, documentation forms, and assessment tools. It prioritizes seven evidence-based evaluation domains: therapeutic efficacy, drug selection, dosing appropriateness, adverse reactions, drug-drug/food interactions, cost-effectiveness, and medication adherence. By integrating practical workflows with clinical decision-support tools, the consensus aims to optimize therapeutic outcomes, mitigate safety risks, and provide actionable guidance for healthcare professionals managing this high-risk population

## 1. Introduction

Elevated low-density lipoprotein cholesterol (LDL-C) constitutes a central pathogenic mechanism driving plaque progression and increased ischemic stroke risk in carotid atherosclerosis with plaque formation 1. Effective cholesterol-lowering therapy demonstrates significant clinical benefits through reducing high-risk plaque features (e.g., hypoechoic morphology, superficial ulceration), decreasing recurrence rates of cerebrovascular events and mortality, while improving patients' quality of life 2, 3. The 2018 U.S. Cholesterol Management Guidelines emphasize multidisciplinary collaboration as an effective strategy for cholesterol optimization, specifically highlighting pharmacist interventions to enhance therapeutic adherence 1. Meta-analyses corroborate that pharmacist-led interventions significantly improve both therapeutic efficacy and medication adherence in lipid-lowering therapies 4, 5. The National Health Commission of China's Guidelines for Accelerating High-Quality Pharmaceutical Services Development (2019) explicitly mandates pharmacists' proactive role in chronic disease management through medication follow-up and therapy optimization. Therefore, pharmacists should actively engage in cholesterol management protocols to ensure medication safety and resolve drug therapy-related issues through professional pharmaceutical care.

Currently, medical community lacks standardized pharmaceutical care protocols for cholesterol-lowering therapy management in patients with carotid atherosclerosis and plaque, while significant disparities in theoretical knowledge and clinical competencies exist among pharmacists across different tiers of healthcare institutions. These limitations impede the standardized, homogeneous implementation of cholesterol-lowering pharmacotherapy management for this patient population. To address this critical gap, the PLA General Hospital has led the development of the Expert consensus on the management of cholesterol-lowering drug therapy in patients with carotid atherosclerosis and plaque disease (hereinafter referred to as "the Consensus"), which underwent rigorous review by pharmaceutical and medical experts from eight nationally representative healthcare institutions.

This Consensus focuses on comprehensive lipid profile optimization and plaque stabilization management in carotid atherosclerosis patients. It systematically outlines standardized pharmaceutical care processes for pharmacists, including baseline data collection, therapeutic regimen analysis, individualized intervention implementation, and longitudinal therapeutic monitoring. The drafting committee structured the Consensus around medication therapy management (MTM) workflows, specifically addressing clinical challenges in evaluating cholesterol-lowering regimens, such as efficacy assessment, drug selection criteria, dosage optimization, adverse effect surveillance, and drug interaction management.

The development process incorporated extensive literature reviews of current international guidelines, recent expert consensus statements, global prescribing information, and authoritative pharmacotherapy databases (UpToDate®, Lexicomp®). Clinical recommendations were further contextualized with China's healthcare policies, including the National Essential Medicines Policy, National Centralized Drug Procurement Policy, and National Medical Insurance Drug Negotiation Policy. By integrating evidence-based medicine with practical clinical experience, this Consensus aims to establish China's first standardized MTM framework for carotid atherosclerosis management. Its implementation is expected to enhance pharmacists' competencies in lipid management and plaque stabilization, optimize therapeutic outcomes through policy-aligned interventions, and reduce medication-related risks through systematic adverse effect monitoring.

### 1.1 Epidemiology

The epidemiological characteristics of carotid atherosclerotic plaques exhibit marked population heterogeneity and regional disparities. The China Kadoorie Biobank (CKB) study reported an average carotid intima-media thickness (cIMT) of 0.70 mm among 24,822 adults across 10 Chinese regions, with a 0.08 mm increase per decade 6. Carotid plaques were detected in 31% of adults, showing a sharp prevalence escalation from 6% in those aged 40-49 to 58% among 70-89-year-olds, alongside interregional variations of 11%-50%. In northeastern China, the crude prevalence reached 42.1% among individuals over 40, with plaques accounting for 40.0% and carotid stenosis at 3.6%, compounded by suboptimal risk factor control (e.g., only 4.7% hypertension management) 7. Compared to European populations, Chinese adults demonstrated thicker cIMT (0.70 vs 0.66 mm, p < 0.001) despite comparable overall plaque burden 6.

Key risk factors included hypertension, elevated LDL-C, diabetes, and smoking (34% plaque prevalence in smokers vs 23% in non-smokers) 6. Blood pressure control emerged as particularly critical, with systolic pressure ≥160 mmHg conferring double the plaque risk compared to < 120 mmHg (39% vs 19%). Notably, young stroke patients (32-50 years) without conventional risk factors still showed significantly elevated cIMT progression versus controls 8. Metabolic analyses revealed positive correlations between insulin resistance and plaque area, while higher BMI displayed paradoxical protective trends 9. High-risk subgroups exhibited stark profiles: 74.3% of stage 3-5 chronic kidney disease patients had detectable plaques, with each 1 pmol/L serum sclerostin increase elevating plaque risk by 2.6% 10. Inflammatory mechanisms showed TLR4 overexpression tripled cerebrovascular event risk, and fibrinogen > 407 mg/dL quintupled thrombotic risk alongside fibrous cap thinning 11.

Geographical variations manifested prominently, with 12.5% moderate-severe stenosis and 76% stable hyperechoic plaques in sub-Saharan African stroke patients, contrasting with China's northern regions demonstrating 31.0% prevalence 6. Gender-specific phenotypes emerged: males predominantly developed structurally complex plaques compared to females' lipid-rich vulnerable lesions. These population-specific patterns underscore the imperative for precision risk factor management and optimized healthcare resource allocation in China.

## 2. Pathophysiological Mechanisms of Carotid Atherosclerotic Plaque

The pathophysiological mechanisms of carotid atherosclerotic plaques involve complex interactions among hemodynamics, inflammatory responses, and vascular remodeling. Below is a systematic synthesis based on current research evidence, as listed in **Table 1**.

### 2.1 Hemodynamics and Endothelial Dysfunction

Atherosclerosis preferentially develops in curved and branching arterial regions, where endothelial cells are exposed to disturbed flow characterized by low-magnitude oscillatory shear stress. Flow dynamics regulate endothelial structure, function, transcriptome, epigenome, and metabolism through mechanosensors and mechanotransduction pathways 12-15. Wall shear stress (WSS) is a critical mechanical factor in plaque formation. Normal laminar shear stress (10-70 dyn/cm²) suppresses inflammation and smooth muscle proliferation via endothelial nitric oxide (NO) pathway activation. In contrast, low/oscillatory shear stress (< 4 dyn/cm²) induces endothelial cell gap widening, increased permeability, and promotes low-density lipoprotein (LDL) infiltration and monocyte adhesion 16-19. Clinical studies demonstrate that hemodynamically significant stenosis (e.g., at the carotid sinus) correlates with increased white matter hyperintensity volume, suggesting synergistic effects of hypoperfusion and endothelial dysfunction 16, 18. Moreover, disturbed flow transmits low-magnitude oscillatory shear stress to endothelial cells, inducing *in situ* reprogramming, which promotes endothelial inflammation, endothelial-to-mesenchymal transition, endothelial-to-immune-like cell transformation, and metabolic alterations, thereby accelerating atherosclerosis 20, 21.

### 2.2 Inflammatory Response and Plaque Formation

Compared with other arteries, particularly the femoral artery, human carotid atherosclerotic lesions exhibit a higher inflammatory burden, with an increased prevalence of anti-inflammatory foam cell-like macrophages, TREM2-positive macrophages, and LYVE1-positive resident macrophages 22, 23. Under low shear stress, endothelial cells upregulate vascular cell adhesion molecule-1 (VCAM-1) and monocyte chemoattractant protein-1 (MCP-1), promoting macrophage infiltration and foam cell formation. Plaque composition analysis reveals that embolic ischemic patients exhibit higher T-cell density, macrophage area, and matrix metalloproteinase-9 (MMP-9) expression in plaques compared to hemodynamic ischemic patients, indicating intensified inflammation-driven plaque instability 24, 25.

### 2.3 Mechanical Mechanisms of Plaque Instability

High time-averaged wall shear stress (TAWSS > 60 dyn/cm²) is associated with recent symptomatic carotid stenosis, potentially inducing fibrous cap rupture or surface thrombosis via mechanical stress. Computational fluid dynamics (CFD) simulations show that symptomatic plaques exhibit significantly higher local maximum WSS than asymptomatic plaques (116.65 vs. 68.28 dyn/cm²), with reduced relative residence time (RRT), reflecting flow disturbances that exacerbate plaque erosion risk 26, 27. Additionally, mechanical stress influences vascular macrophage biomechanics, enhancing matrix metalloproteinase activity and inducing immediate-early gene responses to mechanical deformation. In human monocytes/macrophages and THP-1 cells, biomechanical strain induces class A scavenger receptor expression, a critical lipoprotein receptor in atherogenesis, further promoting plaque formation 28.

### 2.4 Vascular Remodeling and Hemodynamic Adaptation

Carotid bifurcation geometry influences local flow patterns. Smaller luminal expansion (reduced FlareA) is independently associated with vulnerable plaques (lipid-rich or intraplaque hemorrhage; OR=0.45) 29, 30. Compensatory vascular dilation maintains luminal area in early stages, but persistent low shear stress leads to negative remodeling, characterized by intimal thickening and smooth muscle migration 31, 32. In smooth muscle cells, C-terminal β-catenin signaling promotes vascular remodeling by activating the S1pr1 promoter and inducing S1PR1 expression, facilitating neointimal formation following arterial injury and contributing to atherogenesis 33.

### 2.5 Immune Microenvironment and Hemodynamic Interactions

Alterations in the immune microenvironment are fundamental pathological mechanisms by which external factors influence plaque formation and progression. CFD combined with mass cytometry reveals increased neutrophil infiltration in high oscillatory shear index (OSI) regions, while high WSS regions are dominated by anti-inflammatory M2 macrophages, suggesting a flow-dependent immune polarization that contributes to atherosclerotic plaque progression 34, 35. Additionally, complement system activation exacerbates endothelial inflammation in low shear stress regions, whereas physiological shear stress attenuates this effect by upregulating complement-inhibitory proteins 16, 18.

### 2.6 Clinical Implications and Therapeutic Targets

Plaque stability assessment requires integration of hemodynamic parameters (e.g., WSS, OSI) and morphological features (e.g., luminal expansion, lipid core percentage) 26, 27, 29, 30. Emerging technologies like arterial wall magnetic resonance imaging (MRI) and adaptive pulse wave imaging (PWI) enable simultaneous evaluation of plaque biomechanics and hemodynamic status, guiding personalized treatment 26, 27, 36. Future research should clarify spatiotemporal relationships between local hemodynamics and immune responses to develop precision therapies targeting mechanotransduction pathways 37.

## 3. Classification of plaques

### 3.1 Plaque Classification-By Degree of Stenosis Based on the North American Symptomatic Carotid Endarterectomy)

#### 3.1.1 Trial (NASCET) criteria

1) Mild stenosis (< 50%): Minimal hemodynamic impact, low stroke risk.

2) Moderate stenosis (50%-69%): Requires combined assessment of plaque stability for stroke risk stratification.

3) Severe stenosis (≥ 70%): Significant hemodynamic compromise; elevated stroke risk, particularly with vulnerable plaque features.

### 3.2 By Plaque Stability

Stable plaque: Dominated by calcification and fibrous components with intact fibrous caps. Calcified plaques on CT correlate with reduced symptomatic risk (OR = 0.047, p = 0.03) 38.

Vulnerable plaque: Pathological features include thin fibrous caps, large lipid cores, intraplaque hemorrhage, or neovascularization. Hypoechoic plaques on ultrasonography are strongly associated with stroke risk (sensitivity 92.1%, specificity 95.6%) 39.

### 3.3 By Clinical Presentation

1) Symptomatic plaque: Ipsilateral ischemic events within 6 months necessitate aggressive intervention.

2) Asymptomatic plaque: Annual stroke risk of 0.5%-1.0%, but high-risk features (e.g., rapid progression, microembolic signals on TCD, silent infarcts) warrant escalated management.

## 4. Risk Assessment Framework

### 4.1 Imaging Modalities

1) Ultrasonography: Evaluates stenosis and plaque echogenicity; hypoechoic regions (>40% plaque area) and irregular surfaces predict vulnerability. Machine learning models improve classification accuracy (AUC ≈ 0.95) 40.

2) CT Angiography (CTA): Detects calcification; calcified plaques show a 21-fold lower risk of symptomatic presentation (p = 0.03).

3) Magnetic Resonance Imaging (MRI): High-resolution plaque imaging identifies fibrous cap integrity and intraplaque hemorrhage, achieving 94% specificity for vulnerable plaques 41.

### 4.2 Multimodal Risk Stratification

1) High-risk morphological features: Ulceration, irregular surface, or hypoechoic components.

2) Hemodynamic parameters: Ipsilateral cerebrovascular reserve < 1.5 indicates hypoperfusion, tripling stroke risk 40, 42.

3) Systemic atherosclerotic burden: Carotid plaque score ≥ 2 (bilateral/multiple plaques) correlates with coronary artery calcification (r = 0.62, p < 0.001) 43.

### 4.3 Dynamic Monitoring Strategies

**For asymptomatic stenosis:** Biannual ultrasonography to track plaque progression (annual growth > 15% indicates high risk). Emerging molecular imaging (e.g., VCAM-1-targeted MRI probes) quantifies plaque inflammation to guide statin therapy 44, 45.

### 4.4 Clinical Pathway Recommendations

1) Low-risk plaques (stable with < 50% stenosis): Intensive lifestyle interventions and LDL-C control < 2.6 mmol/L.

2) Intermediate-risk plaques (vulnerable or 50%-69% stenosis): LDL-C target < 1.8 mmol/L with antiplatelet therapy.

3) High-risk plaques (symptomatic/vulnerable with ≥ 70% stenosis): Vascular reconstruction (CEA/CAS) combined with aggressive lipid-lowering (≥ 50% LDL-C reduction).

4) Future Directions: Validate AI-assisted diagnostic systems (e.g., quadruple-path neural networks) for risk stratification, and explore integrated models combining multi-omics biomarkers and imaging features.

## 5. Diagnostic method

### 5.1 Ultrasonography

#### 5.1.1 Conventional Ultrasound (US)

1) Advantages: As a first-line screening tool, US noninvasively evaluates carotid intima-media thickness (IMT) and plaque morphology (location, size, echogenicity). IMT ≥ 1.5 mm defines plaque formation. It is sensitive to calcified plaques (high echogenicity) but limited in differentiating intraplaque hemorrhage (IPH) from lipid-rich necrotic core (LRNC) 46, 47.

2) Clinical Application: Recommended for annual follow-up in patients with mild to moderate stenosis (< 70%). Severe stenosis (≥ 70%) requires further multimodal imaging.

#### 5.1.2 Contrast-Enhanced Ultrasound (CEUS)

1) Advantages: Microbubble contrast agents enhance neovascularization imaging, detecting intraplaque angiogenesis and ulceration with > 80% sensitivity 46, 47.

2) Limitations: Operator-dependent and prone to calcification artifacts; requires validation with complementary techniques.

#### 5.1.3 Intravascular Ultrasound (IVUS)

1) Advantages: Quantifies plaque burden and identifies LRNC (hypoechoic regions) and calcification, though limited in assessing fibrous cap thickness 48, 49.

2) Clinical Application: Used for precise plaque morphology evaluation prior to endovascular interventions.

#### 5.1.4 Transcranial Doppler Ultrasound (TCD)

1) Advantages: Real-time hemodynamic monitoring and microembolic signal (HITS) detection for stroke risk stratification.

2) Limitations: Requires adequate acoustic bone windows; best combined with other imaging modalities.

### 5.2 CT Imaging

#### 5.2.1 Computed Tomography (CT)

1) CT Angiography (CTA)

2) Advantages: High spatial resolution visualizes luminal stenosis, calcified plaques (CT attenuation > 130 HU), and ulceration (depth > 1 mm) 50, 51.

3) Clinical Application: Preferred for evaluating complex vascular anatomy and calcified plaques.

#### 5.2.2 Gemstone Spectral CT (GSCT)

1) Advantages: Differentiates LRNC (rising curve), IPH (attenuated curve), and calcification (descending curve) via spectral analysis, reducing metal artifacts.

2) Limitations: Radiation exposure considerations; ideal for detailed calcification analysis.

### 5.3 Magnetic Resonance Imaging (MRI)

#### 5.3.1 High-Resolution MRI (HR-MRI)

1) Advantages: Superior soft-tissue contrast identifies vulnerable plaque features: thin fibrous cap (FC < 0.7 mm), IPH (T1WI hyperintensity), and LRNC (non-enhancing on T1WI) 52.

2) Clinical Application: Recommended for compositional plaque assessment and risk stratification in symptomatic stenosis.

#### 5.3.2 MR Vessel Wall Imaging (MR-VWI)

1) Advantages: 3D techniques (e.g., 3D-MERGE, SNAP) quantify plaque volume and components, enabling craniocervical joint imaging.

2) Recommendation: Annual monitoring for plaque progression in moderate-to-severe stenosis.

### 5.4 Nuclear Medicine Imaging

**Positron Emission Tomography (PET)**

1) Advantages: 18F-FDG (inflammatory activity) and 18F-NaF (microcalcifications) tracers detect plaque vulnerability. The SCAIL score (integrating stenosis and inflammation) predicts stroke recurrence (specificity 90%) 53.

2) Limitations: High cost; reserved for research or high-risk metabolic profiling.

### 5.5 Digital Subtraction Angiography (DSA)

1) Advantages: Gold standard for comprehensive evaluation of stenosis, collateral circulation, and plaque morphology.

2) Limitations: Invasive risks (e.g., plaque disruption, vascular injury); reserved for pre-interventional planning or complex cases.

### 5.6 Artificial Intelligence (AI)

1) Advantages: Machine learning algorithms (e.g., SNAP-based segmentation) automate plaque component detection (IPH, LRNC, FC), reducing observer variability.

2) Future Directions: Multimodal imaging combined with AI enhances risk stratification and personalized management.

### 5.7 Summary

Diagnostic strategies should integrate clinical needs and technical strengths, as listed in **Table 2**.

1) Screening and Follow-up: Begin with US, supplemented by CTA or MRI for plaque characterization.

2) High-Risk Plaque Evaluation: Prioritize HR-MRI or PET/CT for metabolic and morphologic profiling.

3) Pre-Interventional Planning: Combine IVUS, DSA, and CTA for precision.

## 6. Treatment

### 6.1 General treatment

General management primarily involves lifestyle modifications. A healthy lifestyle includes a balanced diet, regular physical activity, weight control, smoking cessation, and limited alcohol consumption, all of which contribute to reducing cardiovascular risk factors such as hypertension and hyperlipidemia 54, 55. Lifestyle modifications play a crucial role in the prevention and management of carotid atherosclerotic plaques and stroke.

#### 6.1.1 Balanced Diet

A well-structured diet is a key component of lifestyle management. The Mediterranean diet has been shown to reduce the risk of cardiovascular events, particularly stroke. This diet emphasizes the consumption of fresh vegetables and fruits (especially green leafy vegetables), whole grains, and fish (preferably those rich in omega-3 fatty acids). It recommends limited intake of red meat and poultry, substitution of high-fat dairy products with low-fat or skimmed dairy, and the inclusion of olive oil, nuts, and moderate amounts of red wine 56-58. Increasing the intake of whole grains, legumes, and tubers; fresh vegetables and fruits; fish; and moderate amounts of eggs, soy products, dairy, and nuts is beneficial 59-64. Additionally, moderate tea consumption has been associated with cardiovascular benefits 65-67. Reducing saturated fat intake, limiting salt consumption (< 5 g/day), minimizing processed meats, controlling cholesterol intake, and avoiding sugar-sweetened beverages and trans fats contribute to reducing the risk of cardiometabolic diseases 68-70.

A diet primarily based on grains is the foundation of balanced nutrition. General dietary recommendations for the population include daily intake of 250-400 g of grains and tubers, including 50-150 g of whole grains and legumes and 50-100 g of tubers. Daily fresh vegetable intake should be ≥ 500 g, with dark-colored vegetables comprising at least half, and fresh fruit intake should be 200-350 g, as fruit juice cannot replace whole fruit. Weekly fish intake should be ≥300 g, prepared using non-frying methods such as boiling or steaming to preserve nutrients 71.

Daily meat consumption should range between 40-75 g, with limited intake of red meat (e.g., pork, beef, lamb). Egg consumption should be 3-6 per week. For individuals with hypercholesterolemia and those at high cardiovascular risk, daily cholesterol intake should be < 300 mg (approximately the amount in one egg yolk) 72-74. If other high-cholesterol foods such as organ meats, red meat, or shrimp are consumed, egg intake should be reduced accordingly. Other recommendations include a daily soybean intake of 25 g, a weekly nut intake of 50-70 g, and a daily dairy intake of 150-300 g. Sugar-sweetened beverages should be avoided. Moderate tea consumption is recommended, with a monthly tea intake of 50-250 g, preferably green tea, while avoiding excessive consumption of strong tea over long periods.

#### 6.1.2 Moderate Exercise

The detrimental effects of a sedentary lifestyle on health have been well-documented in multiple studies, underscoring the importance of minimizing prolonged sitting 75. Regular and sustained physical activity has been associated with a reduced risk of cardiovascular events. In asymptomatic patients undergoing carotid endarterectomy, consistent exercise serves as a crucial factor in preventing cerebrovascular disease and mortality 76. The type and intensity of exercise should be tailored to individual needs and follow a progressive approach 77. Adults are recommended to engage in brisk walking, jogging, swimming, or cycling, accumulating at least 150 minutes of moderate-intensity or 75 minutes of high-intensity physical activity per week. For older adults, activities such as Tai Chi may be beneficial 78.

#### 6.1.3 Weight Management

Obesity (BMI ≥ 30 kg/m2; Asia: BMI ≥ 27.5 kg/m2) and overweight status (25.0 kg/m2 ≤ BMI < 30 kg/m2; Asia: 23.0 kg/m2 ≤ BMI < 27.5 kg/m2) are associated with an increased risk of cardiovascular and cerebrovascular diseases compared to individuals with normal weight 79. In particular, visceral obesity has been identified as a novel risk factor for atherosclerosis and cardiovascular disease 80. Weight control through caloric restriction and increased physical activity has been shown to mitigate cardiovascular event risk 81. Recommended daily caloric intake for weight management is 6,276-7,530 kJ/day (1,500-1,800 kcal/day) for men and 5,020-6,276 kJ/day (1,200-1,500 kcal/day) for women 82.

### 6.2 Drug treatment

Early studies suggested that surgical intervention provided a significant advantage over medical therapy alone in patients with asymptomatic carotid atherosclerotic plaques 83. However, with advancements in pharmacological treatments, recent evidence indicates that medical therapy is becoming equally effective as vascular reconstruction 84. In patients with asymptomatic carotid stenosis, carotid endarterectomy (CEA) or carotid artery stenting (CAS) does not provide a significant advantage over optimal medical therapy in preventing ischemic stroke within one year 85, nor does it differ in long-term mortality risk 86. Therefore, medical therapy should be implemented in both symptomatic and asymptomatic carotid stenosis 87. The three pillars of pharmacological management include antiplatelet therapy, antihypertensive therapy, and lipid-lowering therapy. All patients diagnosed with carotid atherosclerotic disease should receive antiplatelet agents and lipid-lowering drugs, regardless of their serum cholesterol levels.

#### 6.2.1 Antiplatelet

Antiplatelet therapy inhibits platelet adhesion and aggregation, preventing thrombus formation and mitigating the progression of vascular occlusive disease. Although randomized controlled trials have not confirmed that antiplatelet therapy reduces stroke risk in asymptomatic carotid stenosis, long-term use of low-dose aspirin has been shown to reduce myocardial infarction and vascular mortality. Furthermore, perioperative low-dose aspirin therapy can lower the risk of stroke 87-90. Consequently, all patients with carotid stenosis should receive antiplatelet therapy. Commonly used agents include enteric-coated aspirin, clopidogrel, and ticagrelor. The main adverse effects are gastrointestinal symptoms and an increased risk of bleeding. Some studies have also reported a positive correlation between antiplatelet therapy and a higher incidence of intraplaque hemorrhage (IPH), with prolonged antiplatelet use associated with increased IPH frequency. Furthermore, daily doses exceeding 1.0 mg have been linked to a higher occurrence of IPH 91.

#### 6.2.2 Lipid lowering

Lipid-lowering therapy is the cornerstone of managing carotid atherosclerotic plaques. The protective effects of statins extend beyond patients with hypercholesterolemia 92-94. In a subgroup of 1,007 patients with an average carotid stenosis of 51%, high-intensity lipid-lowering therapy led to a remarkable 33% reduction in the risk of any stroke. Beyond stabilizing plaques, lipid-lowering therapy can potentially "reverse" plaques, reducing their volume, modifying their composition, and suppressing inflammatory responses. This multifaceted approach inhibits the progression of coronary atherosclerosis and reduces cardiovascular events 95-97.

The primary goal of lipid-lowering therapy is to reduce low-density lipoprotein cholesterol (LDL-C) levels. Statins are the mainstay of treatment and should be taken consistently over the long term. All patients with carotid stenosis should receive statin therapy. For patients with unstable plaques or a high risk of stroke, intensive lipid-lowering therapy is recommended, targeting LDL-C levels below 1.8 mmol/L or achieving at least a 50% reduction in LDL-C levels if baseline levels range from 1.8 to 3.5 mmol/L 98. If statin monotherapy is insufficient, ezetimibe or other lipid-lowering agents may be added 99.

The evaluation of cholesterol-lowering drug selection encompasses two critical dimensions: unjustified statin non-use assessment (rationality of avoiding statins) and contraindication assessment (appropriateness of drug selection in cases of contraindications or special physiological states).

##### ① Unjustified Statin Non-Use Assessment

Current international guidelines 100-102 unequivocally designate statins as first-line therapy for carotid atherosclerosis with plaque. Statins should be prioritized and maintained in treatment regimens unless contraindications or intolerance exist. Notably, in patients with severe carotid stenosis (> 70%), high-intensity statin initiation warrants caution regarding intraplaque hemorrhage risk, recommending halved starting doses with neurological symptom monitoring 102.

##### Stating intolerance is defined by meeting all four criteria 103

**a)** Clinical manifestations: Symptomatic adverse reactions or abnormal laboratory markers.

**b)** Drug exposure: Intolerance to ≥ 2 statins, with at least one administered at the minimum daily dose.

**c)** Temporal causality: Adverse events occurring post-initiation/dose escalation, resolving upon discontinuation and recurring upon rechallenge.

**d)** Exclusion of confounders: Ruling out comorbidities, drug interactions, or other causative factors.

For patients failing to achieve LDL-C targets with statin monotherapy, combination therapy with non-statin agents (e.g., cholesterol absorption inhibitors, PCSK9 inhibitors) is recommended. Expected LDL-C reductions are stratified as shown in **Table 3**:

##### Stations: Dose-dependent reductions per Table 3

Cholesterol absorption inhibitors: 15-22% reduction monotherapy; additive 21-27% reduction when combined with statins. PCSK9 inhibitors: 47-57% reduction monotherapy; synergistic 44-75% reduction in combination regimens.

##### ② Contraindication Assessment

Drug selection must account for contraindications and physiological constraints (e.g., pregnancy, hepatic/renal impairment, geriatric status) as detailed in **Table 4.** Therapeutic decisions should integrate patient-specific characteristics and prescribing restrictions from official drug labels.

##### Evaluation Recommendations

Unjustified statin non-use: Identified when statins are omitted despite absence of contraindications or intolerance.

Contraindication violation: Determined when contraindicated agents are prescribed without appropriate clinical justification.

##### Dosage and Administration Evaluation

The evaluation of cholesterol-lowering drug regimens requires comprehensive understanding of dosing parameters including standard/maximum doses, administration frequency, timing, and treatment duration. Key principles include:

##### ① Dosage Compliance

Prescribed doses must adhere to drug labeling specifications, particularly in patients with hepatic or renal impairment (see **Table 5** for dose adjustment criteria).

##### ② Administration Protocol

Short-acting statins (simvastatin, lovastatin, pitavastatin, fluvastatin, pravastatin): Administered at bedtime to align with circadian cholesterol biosynthesis patterns.

Long-acting statins (atorvastatin, rosuvastatin) and extended-release formulations: Fixed daily timing without circadian restriction.

PCSK9 inhibitors: Subcutaneous injection technique requires rotation between the upper arm (lateral aspect), abdomen (> 5 cm from umbilicus), and thigh, avoiding areas of ecchymosis or induration. Post-injection monitoring mandates carotid ultrasound at 1 month to assess initial plaque response.

##### ③ Treatment Duration

Cholesterol-lowering therapies typically require indefinite continuation without self-directed dose reduction or discontinuation.

##### Evaluation Recommendations

Regimens are deemed inappropriate if:

Single dose exceeds labeled maximum thresholds.

Unsupervised dose reduction or therapy cessation occurs.

Administration methods deviate from evidence-based protocols.

##### Adverse Reaction Evaluation

The assessment of adverse reactions in cholesterol-lowering therapy involves identifying and managing both existing and potential medication-related risks to ensure therapeutic safety. Adverse effects associated with lipid-lowering agents, particularly statins, primarily stem from two aspects: ① the intrinsic harm of the reactions themselves and ② overestimation of harm leading to unnecessary treatment cessation. While most patients tolerate cholesterol-lowering therapies well, a minority may experience adverse events requiring prompt identification and intervention. Conversely, excessive concern over adverse effects often results in premature discontinuation, inadvertently increasing the risk of ischemic stroke and carotid plaque progression. Clinical studies indicate that nearly half of patients discontinue statins during long-term treatment, with safety concerns being a key contributing factor 104, 105. A recent meta-analysis of 4 million patients in primary and secondary cardiovascular prevention revealed that true statin intolerance occurs in fewer than 10% of cases 106-108, underscoring the urgent need to address disproportionate patient anxiety regarding medication safety. Common adverse reactions and their clinical features are summarized in **Table 6** 29, 109, while table 6 outlines evidence-based protocols for monitoring and managing statin-related hepatic, muscular, and glycemic safety.

During adverse reaction management, causality assessment is critical and may utilize standardized tools such as the National Medical Products Administration Adverse Reaction Evaluation Criteria 110 or the Statin-Associated Muscle Symptom Clinical Indexto determine the likelihood of drug-related causality. These evaluations inform clinical decision-making by differentiating true adverse drug reactions from coincidental symptoms.

Clinical Recommendations: Adverse reaction assessments should integrate symptomatic manifestations, laboratory findings (e.g., liver enzymes, creatine kinase, glucose levels), and characteristic drug-specific toxicity profiles to guide timely interventions. Clinicians are advised to prioritize patient education to mitigate unwarranted treatment discontinuation while maintaining rigorous biochemical and clinical surveillance aligned with guideline recommendations.

##### Interaction Evaluation

The evaluation of drug-drug and drug-food interactions in cholesterol-lowering therapy focuses on identifying risks of clinically significant pharmacokinetic or pharmacodynamic interactions and providing actionable mitigation strategies. Stains exhibit two predominant interaction mechanisms:

1. Metabolic Inhibition: Concomitant use with CYP3A4 inhibitors (e.g., macrolides, azole antifungals) reduces the metabolism of CYP3A4-dependent statins (simvastatin, lovastatin), leading to elevated systemic exposure and toxicity risks.

**2.** Transporter Inhibition: Drugs inhibiting organic anion-transporting polypeptide 1B1 (OATP1B1), such as cyclosporine or gemfibrozil, impair hepatic statin uptake, increasing plasma concentrations and myopathy risks.

**3.** Cholesterol absorption inhibitors (e.g., ezetimibe) demonstrate limited interactions but require avoidance of gemfibrozil co-administration due to heightened risks of myotoxicity and cholelithiasis. Special caution is warranted for CYP2C19-metabolized statins (e.g., rosuvastatin), which may attenuate clopidogrel's antiplatelet efficacy, elevating stent thrombosis risks. PCSK9 inhibitors currently show no clinically relevant drug-food interactions. Clinically actionable interactions and management recommendations, derived from drug labeling and Lexicomp® database analyses, are summarized in **Table 7.**

##### Assessment Recommendations

• Interaction Evaluation: Deem an interaction clinically significant if inappropriate drug combinations persist without mitigation.

• Cost-Effectiveness Evaluation: Incorporate regional healthcare economics and individualized patient factors (e.g., insurance coverage, out-of-pocket costs) into therapeutic decision-making.

##### Adherence Evaluation

Adherence to cholesterol-lowering therapy critically determines clinical outcomes in patients with carotid atherosclerosis and plaque, particularly influencing stroke risk and plaque stability. Studies demonstrate that a 10% improvement in medication adherence correlates with a 12% reduction in carotid plaque-related ischemic stroke incidence 111-115. However, suboptimal adherence remains prevalent, driven by concerns over adverse drug reactions or asymptomatic dose omissions 105, 116, 117. Adherence assessments are imperative for patients exhibiting inadequate therapeutic responses.

#### 6.2.3 Surgery

Carotid revascularization includes carotid endarterectomy (CEA) and carotid artery stenting (CAS). CEA remains the gold standard surgical treatment for carotid artery disease, significantly reducing mortality and disability rates.

CAS serves as a minimally invasive and effective alternative, with comparable long-term outcomes to CEA 118-122. However, CAS is associated with a lower periprocedural risk 119, 123, 124. The two procedures are complementary, and the decision to pursue revascularization should consider the degree of stenosis, symptom status, and the institution's perioperative risk management capabilities 120, 125, 126. For patients with mild carotid stenosis, medical therapy is recommended (i.e., <50% stenosis in symptomatic patients and <60% in asymptomatic patients). Patients with symptomatic moderate stenosis should be considered for CEA, while those with severe stenosis are advised to undergo CEA. For selected patients aged ≥75 years with ≥60% asymptomatic carotid stenosis, an expected survival of at least five years, and a high stroke risk despite optimal medical therapy, CEA may be recommended following a multidisciplinary evaluation of risks and benefits. CAS may be considered as an alternative to CEA in symptomatic carotid stenosis, particularly in patients younger than 70 years 99, 125, 127.

#### 6.2.4 Traditional Chinese Medicine

Traditional Chinese Medicine (TCM) is widely used in China and many Asian countries for the treatment of carotid atherosclerotic plaques. Numerous studies have demonstrated the efficacy of integrating TCM with Western medicine in managing carotid atherosclerosis. Clinically, TCM may be used either alone or in combination with Western therapies, particularly benefiting patients who are intolerant or resistant to statin therapy, as listed in **Table 8** 128-134.

##### Tongxinluo (TXL)

Tongxinluo (TXL) is a traditional herbal compound comprising ginseng, leech, frankincense, and borneol. The primary active components include paeoniflorin and ginsenosides. TXL inhibits the progression of atherosclerotic plaques and stabilizes existing plaques by enancing macrophage autophagy, suppressing endothelial cell (EC) apoptosis, and restoring mitochondrial function. These mechanisms are critical in the treatment of atherosclerosis 135-137.

##### Bu-Shen-Ning-Xin Decoction (BSNXD)

Bu-Shen-Ning-Xin Decoction (BSNXD) is a traditional Chinese patent medicine used for decades to prevent estrogen deficiency-induced atherosclerosis. Wang et al. reported that BSNXD administration mitigated early atherosclerotic pathological damage by suppressing adhesion molecule expression, upregulating endothelial estrogen receptor β (ERβ) expression, and increasing serum nitric oxide (NO) levels in ovariectomized rabbits. Notably, BSNXD did not affect serum lipid profiles, endometrial thickness, or hepatic lipid deposition. ERβ modulates target gene expression to protect endothelial cells, with its expression in the endothelium associated with atherosclerosis in postmenopausal women 138-140.

##### Liuwei Dihuang (LWDH)

Liuwei Dihuang (LWDH) is a classic herbal formula with over 1,000 years of history, comprising six herbs: Rehmannia root, Cornus officinalis fruit, Dioscorea tuber, Poria cocos sclerotium, Chinese yam rhizome, and Paeonia root bark. LWDH inhibits homocysteine (Hcy)-induced endothelial apoptosis by modulating the GPR30 pathway, significantly reducing plaque formation in postmenopausal atherosclerotic animal models. Additionally, LWDH suppresses DNMT1-dependent ERα methylation, enhancing estrogen receptor α (ERα) expression to prevent atherosclerosis 141-145.

##### Red Yeast Rice (RYR)

Red Yeast Rice (RYR) is a traditional Chinese medicinal product made by fermenting Monascus purpureus with rice. RYR effectively reduces low-density lipoprotein cholesterol (LDL-C) levels and other cardiovascular risk factors, demonstrating efficacy and safety in carotid atherosclerosis management 146-149. Xuezhikang, a red yeast rice extract containing 13 monoterpenoid compounds and other bioactive ingredients, has been evaluated in a meta-analysis of 20 randomized controlled trials. Daily doses of 1200-4800 mg, over 6-168 weeks, were associated with LDL-C reduction of approximately -1.02 mmol/L (range, -1.20 to -0.83), triglyceride reduction by -0.26 mmol/L (-0.35 to -0.17), total cholesterol (TC) reduction by -1.0 mmol/L (-1.23 to -0.77), and HDL-C elevation by 0.07 mmol/L (0.03-0.11). The LDL-C reduction with RYR is comparable to low-dose statins, such as pravastatin 40 mg, simvastatin 10 mg, and lovastatin 20 mg 150, 151. The anti-atherosclerotic effects of Xuezhikang are attributed to lipid-lowering properties, antioxidant activity, inhibition of vascular smooth muscle cell (VSMC) proliferation and migration, plaque formation prevention, and vasodilation 152, 153.

##### Scutellariae Radix-Coptidis Rhizoma (QLYD)

Scutellariae Radix-Coptidis Rhizoma (QLYD) represents a core herbal combination in traditional Chinese medicine (TCM) formulations for the treatment of atherosclerosis. This combination exhibits immunomodulatory, anti-inflammatory, and glucose-lipid metabolism regulatory effects 154-156. Studies have demonstrated that QLYD prevents and treats atherosclerosis by inhibiting inflammation, improving lipid profiles, and reducing plaque area in atherosclerotic mouse models 157-159.

##### Kaempferol

Kaempferol, a naturally occurring flavonoid found in various medicinal herbs, exhibits broad biological activities, including anti-inflammatory, antioxidant, cardioprotective, and neuroprotective effects 160, 161. Epidemiological studies suggest an inverse relationship between dietary kaempferol intake and cardiovascular disease risk 160, 162. In high-fat diet (HFD)-fed ApoE^-/-^ mice, kaempferol improves vascular morphology, lipid profiles, and suppresses inflammatory responses 163-165. Mechanistically, kaempferol mitigates atherosclerosis by inhibiting the Piezo1 channel and Ca²⁺ influx, regulating mitochondrial membrane potential, and reducing CD36-mediated mitochondrial reactive oxygen species (ROS) production. These effects subsequently modulate the NF-κB/MAPK and HO-1/Nrf2 signaling pathways, reducing macrophage inflammation and foam cell formation 166.

##### Paeonol (PAE)

Paeonol (PAE)is a phenolic compound extracted from the cortex or roots of Paeonia lactiflora Pallas, known for its anti-atherosclerotic properties. PAE exerts anti-inflammatory effects by inhibiting ox-LDL-induced miR-21 expression and TNF-α release while restoring ox-LDL-mediated suppression of PTEN expression in rat vascular endothelial cells (VECs) *in vitro* 167. In ApoE^-/-^ mice, PAE significantly suppresses atherosclerotic progression and stabilizes vulnerable plaques by reducing T/B cell infiltration and vascular smooth muscle cell (VSMC) apoptosis 168.

##### 2,3,5,4′-Tetrahydroxystilbene-2-O-β-D-glucoside (TSG)

TSG, extracted from Polygonum multiflorum, reduces atherosclerosis by inhibiting matrix metalloproteinase (MMP) expression and lowering lipid levels, thus preventing plaque development 169.

##### Tanshinone IIA

Tanshinone IIA, a diterpenoid compound derived from Salvia miltiorrhiza (Danshen), is used extensively in TCM for cardiovascular diseases. Tanshinone IIA exhibits antioxidant, anti-inflammatory, and anti-angiogenic properties 170, 171. It mitigates atherosclerosis by inhibiting THP-1 monocyte adhesion to TNF-α-stimulated human vascular endothelial cells, reducing TNF-α-induced fractalkine/CX3CL1 mRNA expression and soluble fractalkine levels. Additionally, Tanshinone IIA suppresses TNF-α-induced NF-κB nuclear translocation, thereby reducing monocyte-endothelial adhesion and exerting anti-inflammatory effects 172, 173.

##### Rheum officinale

Emodin, extracted from Rheum officinale, protects endothelial cells from oxidative injury by downregulating Bid mRNA and caspase-3, -8, and -9 mRNA expression, offering protection against atherosclerosis 174, 175.

##### Scutellarin

Scutellarin, a major bioactive component of Erigeron breviscapus (Vant.), prevents oxidative stress-induced endothelial dysfunction and endothelial cell injury by upregulating superoxide dismutase (SOD) and downregulating NADPH oxidase 4 (Nox4). Scutellarin's antioxidant properties contribute to the prevention of atherosclerosis *in vivo* 176.

##### Quercetin

Quercetin, a naturally occurring antioxidant present in various medicinal herbs, inhibits dendritic cell activation and excessive ROS production by modulating the PI3K/AKT signaling pathway. It mitigates atherosclerosis in high-fructose-fed models 177. Additionally, quercetin activates the Nrf2 pathway by competitively binding to the Arg483 site of KEAP1, inhibiting macrophage pyroptosis and providing protective effects against atherosclerosis 178, 179.

##### Ellagic Acid (EA)

Ellagic Acid (EA) is a naturally occurring phenolic compound found abundantly in medicinal herbs, with the highest concentration in raspberries 180. EA mitigates oxidative stress by reducing plasma and macrophage lipid peroxidation and preventing endothelial inflammation 181-184. Mechanistically, EA inhibits platelet-derived growth factor receptor beta (PDGFR-β) tyrosine phosphorylation, reduces intracellular reactive oxygen species (ROS) production, and suppresses downstream activation of extracellular signal-regulated kinase 1/2 (ERK1/2). This blockade prevents the expression of cyclin D1 in vascular smooth muscle cells (VSMCs), inhibiting their proliferation and alleviating atherosclerosis 185. Additionally, EA protects endothelial cells by inhibiting NADPH oxidase-induced superoxide overproduction, downregulating inducible nitric oxide synthase (iNOS) to suppress nitric oxide (NO) release, enhancing cellular antioxidant defenses, and attenuating oxLDL-induced LOX-1 upregulation and eNOS downregulation.

##### Myricitrin

Myricitrin, a natural flavonoid isolated from the root bark of Myrica cerifera, exhibits potent antioxidant effects. Myricitrin inhibits ROS-induced endothelial cell apoptosis by modulating antioxidant enzyme activity and the expression of apoptosis-related genes, effectively suppressing early atherosclerotic plaque formation. Moreover, myricitrin reduces lipid peroxidation, blocks NO release, and maintains mitochondrial membrane potential, collectively inhibiting endothelial cell apoptosis and reducing lesion size in ApoE-/- mice 186. Additionally, myricitrin preserves endothelial cell integrity by optimizing the balance between pro- and anti-apoptotic proteins, including Bax, Bad, XIAP, cIAP-2, and survivin, thereby protecting against oxLDL-induced apoptosis 187, 188.

##### Catalpol

Catalpol, an active compound extracted from Rehmannia glutinosa, possesses anti-inflammatory, antioxidant, and insulin-sensitizing properties. Catalpol protects endothelial cells and mitigates atherosclerosis by reducing homocysteine (HCY)-induced endoplasmic reticulum (ER) stress, suppressing NADPH oxidase 4 (NOX4) expression, and blocking the NF-κB/p65 signaling pathway 189. Furthermore, catalpol exhibits anti-inflammatory effects by modulating estrogen receptor alpha (ERα), reducing pro-inflammatory cytokines (C-reactive protein, TNF-α, and IL-1β), and increasing anti-inflammatory cytokine IL-10 levels, thereby attenuating the inflammatory response in atherosclerosis 190-192.

## Funding

Non communicable Chronic Diseases-National Science and Technology Major Project (No. 2024ZD0528200).

## Figures and Tables

**Table 1:** Classification of Carotid Atherosclerotic Plaques and Risk Assessment

Mechanism Category	Key Findings	Clinical Significance
Hemodynamics & Endothelial Dysfunction	1.Low WSS (<4 dyn/cm²) induces endothelial permeability ↑, promoting LDL infiltration 18, 19 2.Hemodynamic stenosis correlates with white matter hyperintensity volume 18	Indicates synergistic effects of hypoperfusion and endothelial dysfunction
Inflammation & Plaque Formation	1.Low WSS upregulates VCAM-1/MCP-1 → macrophage infiltration 25 2.Embolic plaques show T-cell density and MMP-9 expression↑ 25	Highlights inflammatory contribution to plaque instability
Mechanical Plaque Destabilization	High TAWSS (>60 dyn/cm²) correlates with symptomatic stenosis<br>- Symptomatic plaques show higher WSS (116.65 vs 68.28 dyn/cm²) 27	CFD reveals flow disturbance as erosion risk factor
Vascular Remodeling	1.Bifurcation geometry affects flow patterns 2.Reduced FlareA correlates with vulnerable plaques 30	Negative remodeling manifests as intimal thickening
Immune-Hemodynamic Interactions	1.High OSI zones: neutrophil infiltration↑ 2.High WSS zones: M2 macrophage polarization 35 2.Complement activation in low WSS regions 35	Demonstrates flow-pattern dependent immune modulation
Clinical Assessment & Intervention	1.Requires combined WSS/OSI and morphological evaluation 2.Novel MRI/PWI enables simultaneous plaque mechanics-flow assessment 27, 36	Guides personalized therapy; Future focus: mechano-signaling targeted strategies

**Table 2:** Diagnostic Methods for Carotid Atherosclerotic Plaques

Imaging Modality	Technique	Advantages	Limitations	Clinical Applications
**I. Ultrasound**	(1) Conventional US	Non-invasive assessment of IMT (≥1.5mm defines plaque), plaque location/size/echogenicity; sensitive for calcification	Limited differentiation between IPH and LRNC	Annual follow-up for mild-moderate stenosis (<70%); severe stenosis (≥70%) requires combined imaging
	(2) Contrast-Enhanced US (CEUS)	Microbubble-enhanced visualization of neovascularization/ulceration (sensitivity >80%)	Operator-dependent; calcification artifacts	Requires validation with other techniques
	(3) Intravascular US (IVUS)	Quantitative plaque burden assessment; identifies LRNC/calcification	Limited evaluation of fibrous cap thickness	Pre-interventional plaque morphology assessment
	(4) Transcranial Doppler (TCD)	Real-time monitoring of cerebral hemodynamics/microemboli (HITS)	Requires adequate acoustic bone window	Combined use for stroke risk assessment
**II. CT Imaging**	(1) CTA	High spatial resolution for stenosis/calcification/ulcer (depth >1mm); calcification CT value >130 HU	-	Evaluation of complex vascular anatomy and calcified plaques
	(2) Spectral CT (GSCT)	Spectral curves distinguish LRNC (rising), IPH (attenuating), calcification (falling); reduces metal artifacts	Radiation exposure	Detailed analysis of calcified plaques
**III. MRI**	(1) HR-MRI	Superior soft-tissue contrast for vulnerable plaque features: thin fibrous cap (FC<0.7mm), IPH (T1WI hyperintensity), LRNC (T1WI non-enhancement)	-	Plaque composition analysis and risk stratification in symptomatic carotid stenosis
	(2) MR-VWI	3D plaque volume/component quantification (3D-MERGE/SNAP sequences)	-	Annual monitoring for moderate-severe stenosis progression
**IV. Nuclear Imaging**	PET	18F-FDG/18F-NaF detects inflammation/microcalcification; SCAIL score (specificity 90%)	High cost	Research/high-risk patient metabolic evaluation
**V. DSA**	DSA	Gold standard for stenosis/collateral circulation/plaque morphology	Invasive risks (plaque dislodgement, vascular injury)	Pre-interventional assessment or complex cases
**VI. AI Technology**	Machine Learning Algorithms	Automated plaque segmentation (SNAP sequences) for IPH/LRNC/FC identification; reduces human error	-	Multimodal imaging integration improves risk assessment efficiency and personalized care

**Table 3:** Expected LDL-C Reduction Rates of Different Types and Doses of Statins

Drug class	Low intensity	Moderate intensity	High intensity
Amount of LDL-Ca lowering	< 30%	30 to 49%	≥ 50%
Atorvastatin	-	10 to 20 mg	40 to 80 mg
Rosuvastatin	-	5 to 10 mg	20 to 40 mg
Lovastatin	20 mg	40 to 80 mg	-
Pravastatin	20 mg	40 to 80 mg	-
Fluvastatin	40 mg	80 mg	-
Simvastatin	10 mg	20 to 40 mg	-
Pitavastatin	1	1 to 4 mg	-

^a^LCL-C: low-density lipoprotein cholesterol; "-" indicates no available data.

**Table 4:** drugs under different pathophysiological conditions

Drug class	Children(age/year)		Pregnancy	Lactation	Elderly	Hepatic Impairment	Renal Impairment		
	<10	>10					Mild	Moderate	Severe
Atorvastatin	△	√	×	×	√	×#	√	√	√
Rosuvastatin	△	√	×	×	√	×#	√	√	×
Lovastatin	△	√ (< 40 mg/day)	×	×	√	×#	√	√	△
Pravastatin	△	√ (< 40 mg/day)	×	×	√	×#	√	√	△
Fluvastatin	△	√	×	×	√	×#	√	√	×
Simvastatin	△	√	×	×	√	×#	√	√	△
Pitavastatin	△	√	×	×	√	×#	√	△	△
Ezetimibe	△	√	△	△	√	×#	√	√	√
Alirocumab	√	√	△	×	√	△*	√	√	△*
Evolocumab	△	△	△	×	√	△*	√	√	√

×: Contraindicated; △: Use with caution; requires thorough risk-benefit assessment by physicians and careful review of prescribing information; √: Safe for routine use; #: Contraindicated in active liver disease, decompensated cirrhosis, acute liver failure, or unexplained persistent elevation of transaminases (≥3× upper limit of normal); *: Limited data in severe hepatic impairment; use with caution; +: Limited data in corresponding degrees of renal impairment; use with caution.

**Table 5:** Dosing and drug Administration of lipid-lowering drugs

Drug class	Typical Dose		Maximum Dose		Administration	Food Effect
	Adult	Children (aged ≥10 years)	Adult	Children (aged ≥10 years)		
Atorvastatin	10 to 20 mg/d	5 to 10 mg/d	80 mg/d	20 mg/d	Take any time	None
Rosuvastatin	5 to 10 mg/d	5 mg/d	20 mg/d	20 mg/d	Take any time	None
Lovastatin	40 mg/d	10 mg/d	80 mg/d	40 mg/d	With dinner	Increased absorption
Pravastatin	40 mg/d	10 mg/d	40 mg/d	40 mg/d (aged 14 to 18 years) 20 mg/d (aged 8 to 13 years)	At bedtime	absorption
Fluvastatin	80 mg/d	20 mg/d	80 mg/d	80 mg/d	At bedtime	Negligible
Simvastatin	20 to 40 mg/d	10 mg/d (aged ≥10 years) 5 mg/d (aged 10 years)	80 mg/d	40 mg/d	At bedtime	None
Pitavastatin	2 to 4 mg/d	-	4 mg/d	-	Take any time	Decreases
Ezetimibe	10 mg/d	10 mg/d	10 mg/d	10 mg/d	Take any time	None
Alirocumab	75mg, every 2 weeks	-	150 mg, every 2 weeks or 300 mg, every 4 weeks	-	subcutaneous injection	None
Evolocumab	140 mg, every 2 weeks	-	420 mg, every 4 weeks	-	subcutaneous injection	None

**Table 6:** Major side effects of lipid-lowering drugs

Drug class	Major side effects
Statins	Musculoskeletal complications (frequency varies by agent): Myalgia (most common); Myopathy (1-5% in clinical trials); Rhabdomyolysis (rare, <0.1%); Hepatocellular effects: Asymptomatic transaminase elevation (ALT/AST >3× ULN in 0.5-2%); Alkaline phosphatase increase (dose-dependent); Gastrointestinal disturbances: Nausea, diarrhea (5-10%); Constipation, dyspepsia. Neurologic manifestations: Headache (2-5%); Sleep pattern disruption.
Cholesterol absorption inhibitor	Clinical manifestations: Typically, mild and transient, including: Headache (incidence 5-8%); Asthenia/fatigue (3-6%); Gastrointestinal disturbances (nausea, diarrhea; 4-7%); Myalgia (2-4%); Asymptomatic transaminase elevation (ALT/AST >1.5× ULN in 1-3%). Overall adverse event rates were generally comparable to placebo groups in clinical trials (p>0.05)
PCSK9 inhibitors	Characteristic reactions: Injection-site reactions (pain, erythema, pruritus; 10-15%); Systemic flu-like symptoms (myalgia, pyrexia; 5-8%); Hypersensitivity manifestations (rash, urticaria; <1%). Frequency and severity of adverse effects demonstrated non-inferiority to placebo controls across Phase III studies
Adenosine triphosphate citrate lyase inhibitor	Hyperuricemia: May require serum uric acid monitoring (≥7.0 mg/dL in 3-8% of cases). Myalgia/Arthralgia: Mild-to-moderate muscle pain (5-12% incidence); Joint stiffness (3-7%), typically dose-dependent. Muscle spasms: Predominantly nocturnal (2-4%). Tendon rupture: Rare but serious (<0.3%), predominantly affecting Achilles tendon. Aspartate aminotransferase (AST) elevation: ≥3× ULN in 1.5-4% of patients; Requires monitoring (baseline + q12 weeks).

**Table 7:** Common clinically significant drug/food interactions with cholesterol-lowering drugs

Drug / Food	Atorvastatin	Rosuvastatin	Lovastatin	Pravastatin	Fluvastatin	Simvastatin	Pitavastatin	Ezetimibe
Antimicrobials								
Erythromycin	√	√	×	(40)	√	×	(1)	√
Itraconazole	(20)	√	×	√	√	×	√	√
Ketoconazole	√	√	×	√	√	×	√	√
Fluconazole	√	√	√	√	(40)	√	○	√
Rifampin/Rifampicin	√	○	√	√	√	√	(2)	√
Clarithromycin	(20)	√	×	(40)	√	×	○	√
Voriconazole	√	√	×	√	√	×	√	√
Fusidic acid		×	×	×	×	×	×	√
Posaconazole		√	×	√	√	×	√	√
Daptomycin	√	√	√	√	√	×	√	√
Cardiovascular Agents								
Verapamil	○	√	(20)	√	√	(10)	√	√
Amiodarone	√	√	(40)	√	√	(20)	√	√
Gemfibrozil	×	×	×	×	×	×	×	×
Diltiazem	○	√	(20)	√	√	(10)	√	√
Dronedarone	○	√	(20)	√	√	(10)	√	√
Amlodipine	○	√	○	√	√	(10)	√	√
Other Drugs								
Danazol	√	√	(20)	√	√	×	√	√
Regorafenib	√	(5)	√	√	√	√	√	√
Cyclosporine	×	×	×	(20)	(40)	×	×	√
Darolutamide	√	×	√	√	√	√	√	√
Foods								
Grapefruit juice	[1.2]	√	×	√	√	×	√	√
Red yeast rice	×	×	×	×	×	×	×	√

×Contraindicated: Avoid concomitant use.√Standard-dose combination allowed: No dose adjustment required.( )Maximum statin dose when combined: [value] mg/day (e.g., "Simvastatin (20)" indicates 20 mg/day maximum).[ ] Maximum grapefruit juice intake: [value] L/day (applies to statins metabolized by CYP3A4).○Use with caution: Requires thorough risk-benefit assessment by physicians and strict adherence to prescribing information.

**Table 8:** Role of Traditional Chinese Medicine in Carotid Atherosclerotic Plaque Management

Extracts/monomers	Detail mechanisms	Refs.
TXL	Enhancing Macrophage Autophagy, Inhibiting Endothelial Cell (EC) Apoptosis, and Restoring Mitochondrial Function	135, 136
BSNXD	Inhibition of Adhesion Molecule Expression, Upregulation of Endothelial Estrogen Receptor (ER)β Expression, and Increased Nitric Oxide (NO) Levels	138
LWDH	Inhibition of Homocysteine (Hcy)-Induced Endothelial Cell Apoptosis; Upregulation of Estrogen Receptor Alpha (ERα) Expression	141, 142
RYR/Xuezhikang	Lipid-Lowering, Antioxidant Activity, Inhibition of Smooth Muscle Cell Proliferation and Migration, Prevention of Plaque Formation, and Vasodilation	146, 150, 152
QLYD	Inhibition of Inflammation, Improvement of Lipid Profiles, and Reduction of Atherosclerotic Plaque Size in Mouse Models	154, 155, 157
Kaempferol	Inhibition of Inflammation; Regulation of CD36 Expression, Mitochondrial Membrane Potential, ROS Production, NF-κB/MAPK Pathway, and Ca²⁺ Influx; Upregulation of HO-1/Nrf2; Reduction of Macrophage Inflammation and Foam Cell Formation	162, 163, 166
Paeonol	Anti-Inflammatory Effects; Reduction of T/B Cell Infiltration and VSMC Apoptosis	167, 168
2.2,3,4 cent,5-Tetrahydroxystilbene-2-O-beta-D-glucoside	Inhibition of Matrix Metalloproteinase (MMP) Expression	169
Tanshinone IIA	Inhibition of Monocyte Adhesion to Endothelial Cells; Anti-Inflammatory Effects	172
*Rheum officinale*	Protection of Endothelial Cells from Oxidative Damage	174
Scutellarin	Upregulation of Superoxide Dismutase (SOD) and Downregulation of NADPH Oxidase 4 (Nox4), Preventing Oxidative Stress-Induced Endothelial Dysfunction and Damage	176
Quercetin	Inhibition of Dendritic Cell Activation and Excessive ROS Production; Inhibition of Macrophage Pyroptosis	177, 178
Ellagic acid	Inhibition of VSMC Proliferation and Reduction of oxLDL-Induced Endothelial Dysfunction	185
Myricitrin	Inhibition of Endothelial Cell Apoptosis	186, 187
Catalpol	Endothelial Protection and Reduction of Inflammatory Response in Atherosclerosis	189, 190
